# 2-[(*E*)-(2,4-Dichloro­benzyl­idene)amino]­isoindoline-1,3-dione

**DOI:** 10.1107/S1600536811023063

**Published:** 2011-06-18

**Authors:** Mohammad Asad, Chuan-Wei Oo, Hasnah Osman, Madhukar Hemamalini, Hoong-Kun Fun

**Affiliations:** a School of Chemical Sciences, Universiti Sains Malaysia, 11800 USM, Penang, Malaysia; b X-ray Crystallography Unit, School of Physics, Universiti Sains Malaysia, 11800 USM, Penang, Malaysia

## Abstract

In the title compound, C_15_H_8_Cl_2_N_2_O_2_, the mol­ecule adopts an *E* configuration about the central C=N double bond. The isoindoline ring is essentially planar, with a maximum deviation of 0.019 (2) Å. The dihedral angle between the isoindoline ring and the dichloro-substituted benzene ring is 6.54 (9)°. An intra­molecular C—H⋯O hydrogen bond occurs. A short Cl⋯Cl contact of 3.4027 (9) Å is present in the crystal structure. The crystal packing is further stabilized by weak C—H⋯π inter­actions.

## Related literature

For the coordination ability and biological activity of Schiff bases, see: Bhunora *et al.* (2011 ▶); Gupta & Sutar (2008 ▶); Sridhar *et al.*, (2001 ▶); Mladenova *et al.* (2002 ▶); Bharti *et al.* (2010 ▶); Tenorio *et al.* (2005 ▶); Liu *et al.* (1992 ▶); Hodnett & Dunn (1970 ▶).
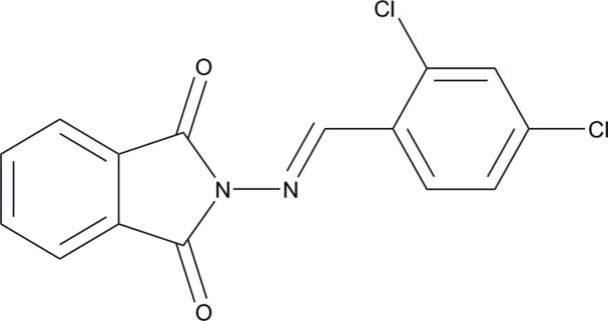


## Experimental

### 

#### Crystal data

C_15_H_8_Cl_2_N_2_O_2_
*M* *_r_* = 319.13
Monoclinic, 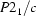

*a* = 8.0387 (8) Å
*b* = 7.6981 (8) Å
*c* = 22.2686 (19) Å
β = 101.828 (3)°
*V* = 1348.8 (2) Å^3^
*Z* = 4
Mo *K*α radiation
μ = 0.49 mm^−1^
*T* = 296 K
0.44 × 0.19 × 0.15 mm

#### Data collection

Bruker APEXII DUO CCD area-detector diffractometer
Absorption correction: multi-scan (*SADABS*; Bruker, 2009 ▶) *T*_min_ = 0.815, *T*_max_ = 0.930
13900 measured reflections
3902 independent reflections
2795 reflections with *I* > 2σ(*I*)
*R*_int_ = 0.033

#### Refinement

*R*[*F* ^2^ > 2σ(*F* ^2^)] = 0.046
*wR*(*F* ^2^) = 0.113
*S* = 1.07
3902 reflections
190 parameters
H-atom parameters constrained
Δρ_max_ = 0.30 e Å^−3^
Δρ_min_ = −0.42 e Å^−3^

### 

Data collection: *APEX2* (Bruker, 2009 ▶); cell refinement: *SAINT* (Bruker, 2009 ▶); data reduction: *SAINT*; program(s) used to solve structure: *SHELXTL* (Sheldrick, 2008 ▶); program(s) used to refine structure: *SHELXTL*; molecular graphics: *SHELXTL*; software used to prepare material for publication: *SHELXTL* and *PLATON* (Spek, 2009 ▶).

## Supplementary Material

Crystal structure: contains datablock(s) global, I. DOI: 10.1107/S1600536811023063/rz2609sup1.cif

Structure factors: contains datablock(s) I. DOI: 10.1107/S1600536811023063/rz2609Isup2.hkl

Supplementary material file. DOI: 10.1107/S1600536811023063/rz2609Isup3.cml

Additional supplementary materials:  crystallographic information; 3D view; checkCIF report

## Figures and Tables

**Table 1:** Hydrogen-bond geometry (Å, °) *Cg*1 is the centroid of the C1–C6 ring.

*D*—H⋯*A*	*D*—H	H⋯*A*	*D*⋯*A*	*D*—H⋯*A*
C7—H7*A*⋯O1	0.93	2.18	2.857 (2)	129
C2—H2*A*⋯*Cg*1^i^	0.93	2.81	3.663 (2)	153

Symmetry code: (i) 
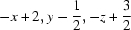
.

**Fig. 1.**
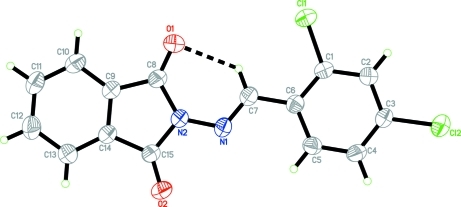
The asymmetric unit of the title compound, showing 30% probability displacement ellipsoids. The intramolecular C—H···O hydrogen bond is shown as a dashed line.

**Fig. 2.**
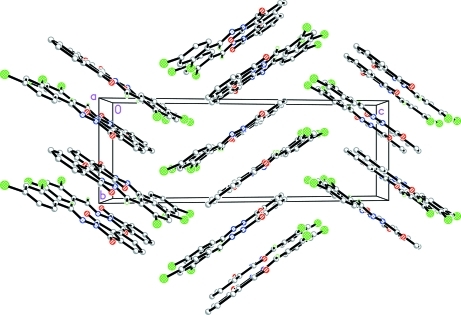
The crystal packing of the title compound viewed down the a axis. Hydrogen atoms are omitted for clarity.

## supplementary crystallographic information

### Comment

Schiff bases have been prepared from the condensation of aldehydes or ketones with amines and their coordination ability with metal ions such as copper (II), cobalt (II), iron (II) and zinc (II) (Bhunora *et al.*, 2011; Gupta & Sutar, 2008) has been studied. Many Schiff bases have been reported to possess antibacterial (Sridhar *et al.,*, 2001; Mladenova *et al.*, 2002); antifungal (Bharti *et al.*, 2010; Tenorio *et al.*, 2005); and antitumor activities (Liu *et al.*, 1992; Hodnett & Dunn, 1970).

In the title compound (Fig. 1), the molecule adopts an *E* configuration about the central C7═N1 double bond.  The isoindoline ring is essentially planar, with a maximum deviation of 0.019 (2) Å for atom C8. The dihedral angle between the isoindoline ring and the dichloro-substituted phenyl ring is 6.54 (9)°.  An intramolecular C—H···O hydrogen bond which generates an *S*(6) ring motif and a short Cl···Cl contact of  3.4027 (9) Å are present in the crystal structure. The crystal packing (Fig. 2) is further stabilized by a weak C—H···π (Table 1) interactions involving the C1–C6 ring.

### Experimental

A mixture of 2-amino-1,3-isoindolinedione (6.15 mmol, 1.0 g) and 2,4-dichloro benzaldehyde (6.15 mmol, 1.08 g) was dissolved in an appropriate amount of ethanol-glacial acetic acid (2:1 *v*/*v*). The mixture was refluxed on water-bath for 0.5 hr to give a white colour precipitate. The solution was cooled at room temperature, filtered off, washed with ethanol and dried. The isolated product was recrystallized from chloroform-methanol (1:1 *v*/*v*)to get the new Schiff base in 80% yield.

### Refinement

All hydrogen atoms were positioned geometrically [C–H = 0.93 Å] and were refined using a riding model, with *U*_iso_(H) = 1.2 *U*_eq_(C).

### Crystal data

**Table:**

C_15_H_8_Cl_2_N_2_O_2_	*F*(000) = 648
*M**_r_* = 319.13	*D*_x_ = 1.572 Mg m^−^^3^
Monoclinic, *P*2_1_/*c*	Mo *K*α radiation, λ = 0.71073 Å
Hall symbol: -P 2ybc	Cell parameters from 4526 reflections
*a* = 8.0387 (8) Å	θ = 2.8–29.8°
*b* = 7.6981 (8) Å	µ = 0.49 mm^−^^1^
*c* = 22.2686 (19) Å	*T* = 296 K
β = 101.828 (3)°	Block, colourless
*V* = 1348.8 (2) Å^3^	0.44 × 0.19 × 0.15 mm
*Z* = 4	

### Data collection

**Table:**

Bruker APEXII DUO CCD area-detector diffractometer	3902 independent reflections
Radiation source: fine-focus sealed tube	2795 reflections with *I* > 2σ(*I*)
graphite	*R*_int_ = 0.033
φ and ω scans	θ_max_ = 30.0°, θ_min_ = 1.9°
Absorption correction: multi-scan (*SADABS*; Bruker, 2009)	*h* = −10→11
*T*_min_ = 0.815, *T*_max_ = 0.930	*k* = −10→10
13900 measured reflections	*l* = −31→29

### Refinement

**Table:**

Refinement on *F*^2^	Primary atom site location: structure-invariant direct methods
Least-squares matrix: full	Secondary atom site location: difference Fourier map
*R*[*F*^2^ > 2σ(*F*^2^)] = 0.046	Hydrogen site location: inferred from neighbouring sites
*wR*(*F*^2^) = 0.113	H-atom parameters constrained
*S* = 1.07	*w* = 1/[σ^2^(*F*_o_^2^) + (0.0331*P*)^2^ + 0.9203*P*] where *P* = (*F*_o_^2^ + 2*F*_c_^2^)/3
3902 reflections	(Δ/σ)_max_ = 0.001
190 parameters	Δρ_max_ = 0.30 e Å^−^^3^
0 restraints	Δρ_min_ = −0.42 e Å^−^^3^

### Special details

**Table:**

Geometry. All esds (except the esd in the dihedral angle between two l.s. planes) are estimated using the full covariance matrix. The cell esds are taken into account individually in the estimation of esds in distances, angles and torsion angles; correlations between esds in cell parameters are only used when they are defined by crystal symmetry. An approximate (isotropic) treatment of cell esds is used for estimating esds involving l.s. planes.
Refinement. Refinement of F^2^ against ALL reflections. The weighted R-factor wR and goodness of fit S are based on F^2^, conventional R-factors R are based on F, with F set to zero for negative F^2^. The threshold expression of F^2^ > 2sigma(F^2^) is used only for calculating R-factors(gt) etc. and is not relevant to the choice of reflections for refinement. R-factors based on F^2^ are statistically about twice as large as those based on F, and R- factors based on ALL data will be even larger.

### Fractional atomic coordinates and isotropic or equivalent isotropic displacement parameters (Å^2^)

**Table:**

	*x*	*y*	*z*	*U*_iso_*/*U*_eq_	
Cl1	0.72877 (6)	−0.18254 (8)	0.79982 (3)	0.05067 (17)	
Cl2	1.31606 (7)	−0.18842 (9)	0.72366 (3)	0.05889 (19)	
O1	0.58896 (19)	0.1209 (2)	0.93934 (8)	0.0566 (5)	
O2	1.08478 (17)	0.3584 (2)	1.04839 (7)	0.0457 (4)	
N1	0.9657 (2)	0.1599 (2)	0.94538 (8)	0.0374 (4)	
N2	0.86224 (19)	0.2180 (2)	0.98379 (7)	0.0347 (4)	
C1	0.9387 (2)	−0.1149 (3)	0.81141 (9)	0.0337 (4)	
C2	1.0327 (2)	−0.1710 (3)	0.76903 (9)	0.0366 (4)	
H2A	0.9837	−0.2409	0.7361	0.044*	
C3	1.2005 (2)	−0.1199 (3)	0.77727 (9)	0.0369 (4)	
C4	1.2766 (2)	−0.0181 (3)	0.82651 (9)	0.0387 (4)	
H4A	1.3906	0.0127	0.8319	0.046*	
C5	1.1798 (2)	0.0371 (3)	0.86757 (9)	0.0367 (4)	
H5A	1.2299	0.1064	0.9005	0.044*	
C6	1.0085 (2)	−0.0087 (3)	0.86084 (8)	0.0330 (4)	
C7	0.9041 (2)	0.0526 (3)	0.90345 (9)	0.0378 (4)	
H7A	0.7932	0.0130	0.8997	0.045*	
C8	0.6843 (2)	0.2004 (3)	0.97890 (9)	0.0366 (4)	
C9	0.6452 (2)	0.3000 (3)	1.03100 (9)	0.0339 (4)	
C10	0.4911 (3)	0.3240 (3)	1.04859 (10)	0.0413 (5)	
H10A	0.3918	0.2728	1.0271	0.050*	
C11	0.4911 (3)	0.4273 (3)	1.09946 (10)	0.0449 (5)	
H11A	0.3897	0.4458	1.1125	0.054*	
C12	0.6395 (3)	0.5040 (3)	1.13147 (10)	0.0491 (5)	
H12A	0.6356	0.5733	1.1654	0.059*	
C13	0.7941 (3)	0.4787 (3)	1.11370 (9)	0.0436 (5)	
H13A	0.8937	0.5295	1.1351	0.052*	
C14	0.7933 (2)	0.3755 (3)	1.06312 (9)	0.0336 (4)	
C15	0.9360 (2)	0.3247 (3)	1.03399 (9)	0.0338 (4)	

### Atomic displacement parameters (Å^2^)

**Table:**

	*U*^11^	*U*^22^	*U*^33^	*U*^12^	*U*^13^	*U*^23^
Cl1	0.0330 (2)	0.0650 (4)	0.0558 (3)	−0.0135 (2)	0.0131 (2)	−0.0105 (3)
Cl2	0.0444 (3)	0.0766 (5)	0.0621 (4)	−0.0071 (3)	0.0259 (3)	−0.0224 (3)
O1	0.0355 (8)	0.0710 (12)	0.0616 (10)	−0.0063 (8)	0.0057 (7)	−0.0274 (9)
O2	0.0290 (7)	0.0587 (10)	0.0490 (9)	−0.0036 (7)	0.0069 (6)	−0.0061 (7)
N1	0.0326 (8)	0.0425 (10)	0.0388 (9)	0.0032 (7)	0.0114 (7)	−0.0030 (7)
N2	0.0294 (7)	0.0386 (9)	0.0368 (8)	0.0007 (7)	0.0082 (6)	−0.0035 (7)
C1	0.0275 (8)	0.0357 (10)	0.0377 (10)	−0.0017 (7)	0.0060 (7)	0.0023 (8)
C2	0.0338 (9)	0.0388 (11)	0.0370 (10)	−0.0015 (8)	0.0066 (8)	−0.0031 (9)
C3	0.0322 (9)	0.0411 (11)	0.0394 (10)	0.0022 (8)	0.0123 (8)	−0.0018 (9)
C4	0.0279 (9)	0.0432 (12)	0.0446 (11)	−0.0031 (8)	0.0068 (8)	−0.0020 (9)
C5	0.0336 (9)	0.0387 (11)	0.0364 (10)	−0.0007 (8)	0.0036 (7)	−0.0033 (8)
C6	0.0319 (9)	0.0342 (10)	0.0331 (9)	0.0018 (8)	0.0071 (7)	0.0024 (8)
C7	0.0329 (9)	0.0434 (12)	0.0383 (10)	0.0007 (8)	0.0101 (8)	−0.0014 (9)
C8	0.0293 (9)	0.0395 (11)	0.0408 (10)	0.0015 (8)	0.0065 (8)	−0.0010 (9)
C9	0.0301 (9)	0.0350 (11)	0.0373 (10)	0.0013 (8)	0.0082 (7)	0.0027 (8)
C10	0.0322 (9)	0.0433 (12)	0.0499 (12)	−0.0011 (9)	0.0116 (8)	0.0036 (10)
C11	0.0407 (11)	0.0473 (13)	0.0517 (12)	0.0051 (9)	0.0216 (9)	0.0036 (10)
C12	0.0541 (13)	0.0529 (14)	0.0445 (12)	0.0032 (11)	0.0198 (10)	−0.0054 (10)
C13	0.0411 (11)	0.0498 (13)	0.0401 (11)	−0.0012 (10)	0.0084 (8)	−0.0066 (10)
C14	0.0309 (9)	0.0348 (10)	0.0354 (9)	0.0017 (8)	0.0077 (7)	0.0029 (8)
C15	0.0295 (8)	0.0372 (11)	0.0348 (9)	−0.0003 (8)	0.0071 (7)	0.0015 (8)

### Geometric parameters (Å, °)

**Table:**

Cl1—C1	1.7338 (19)	C5—H5A	0.9300
Cl2—C3	1.7383 (19)	C6—C7	1.467 (3)
O1—C8	1.209 (2)	C7—H7A	0.9300
O2—C15	1.201 (2)	C8—C9	1.477 (3)
N1—C7	1.268 (3)	C9—C14	1.385 (3)
N1—N2	1.384 (2)	C9—C10	1.386 (3)
N2—C15	1.416 (3)	C10—C11	1.384 (3)
N2—C8	1.419 (2)	C10—H10A	0.9300
C1—C2	1.393 (3)	C11—C12	1.390 (3)
C1—C6	1.393 (3)	C11—H11A	0.9300
C2—C3	1.381 (3)	C12—C13	1.393 (3)
C2—H2A	0.9300	C12—H12A	0.9300
C3—C4	1.384 (3)	C13—C14	1.377 (3)
C4—C5	1.384 (3)	C13—H13A	0.9300
C4—H4A	0.9300	C14—C15	1.482 (3)
C5—C6	1.399 (3)		
			
C7—N1—N2	118.18 (16)	O1—C8—N2	125.65 (18)
N1—N2—C15	117.91 (15)	O1—C8—C9	129.07 (18)
N1—N2—C8	130.24 (16)	N2—C8—C9	105.28 (16)
C15—N2—C8	111.63 (15)	C14—C9—C10	121.44 (19)
C2—C1—C6	122.02 (17)	C14—C9—C8	108.96 (16)
C2—C1—Cl1	116.91 (15)	C10—C9—C8	129.59 (19)
C6—C1—Cl1	121.07 (15)	C11—C10—C9	117.2 (2)
C3—C2—C1	118.21 (18)	C11—C10—H10A	121.4
C3—C2—H2A	120.9	C9—C10—H10A	121.4
C1—C2—H2A	120.9	C10—C11—C12	121.30 (19)
C2—C3—C4	121.86 (18)	C10—C11—H11A	119.3
C2—C3—Cl2	117.88 (16)	C12—C11—H11A	119.3
C4—C3—Cl2	120.26 (15)	C11—C12—C13	121.2 (2)
C5—C4—C3	118.67 (18)	C11—C12—H12A	119.4
C5—C4—H4A	120.7	C13—C12—H12A	119.4
C3—C4—H4A	120.7	C14—C13—C12	117.3 (2)
C4—C5—C6	121.77 (18)	C14—C13—H13A	121.4
C4—C5—H5A	119.1	C12—C13—H13A	121.4
C6—C5—H5A	119.1	C13—C14—C9	121.60 (18)
C1—C6—C5	117.44 (17)	C13—C14—C15	129.51 (18)
C1—C6—C7	120.55 (17)	C9—C14—C15	108.90 (17)
C5—C6—C7	122.01 (18)	O2—C15—N2	124.69 (17)
N1—C7—C6	119.80 (18)	O2—C15—C14	130.08 (19)
N1—C7—H7A	120.1	N2—C15—C14	105.21 (15)
C6—C7—H7A	120.1		
			
C7—N1—N2—C15	174.19 (18)	N2—C8—C9—C14	1.7 (2)
C7—N1—N2—C8	−11.8 (3)	O1—C8—C9—C10	1.8 (4)
C6—C1—C2—C3	0.6 (3)	N2—C8—C9—C10	−178.9 (2)
Cl1—C1—C2—C3	−179.66 (16)	C14—C9—C10—C11	0.3 (3)
C1—C2—C3—C4	1.0 (3)	C8—C9—C10—C11	−179.0 (2)
C1—C2—C3—Cl2	−179.27 (16)	C9—C10—C11—C12	0.1 (3)
C2—C3—C4—C5	−1.6 (3)	C10—C11—C12—C13	−0.4 (4)
Cl2—C3—C4—C5	178.68 (17)	C11—C12—C13—C14	0.2 (4)
C3—C4—C5—C6	0.6 (3)	C12—C13—C14—C9	0.2 (3)
C2—C1—C6—C5	−1.5 (3)	C12—C13—C14—C15	−179.5 (2)
Cl1—C1—C6—C5	178.73 (15)	C10—C9—C14—C13	−0.5 (3)
C2—C1—C6—C7	177.96 (19)	C8—C9—C14—C13	178.99 (19)
Cl1—C1—C6—C7	−1.8 (3)	C10—C9—C14—C15	179.29 (19)
C4—C5—C6—C1	0.9 (3)	C8—C9—C14—C15	−1.3 (2)
C4—C5—C6—C7	−178.58 (19)	N1—N2—C15—O2	−5.7 (3)
N2—N1—C7—C6	178.49 (17)	C8—N2—C15—O2	179.2 (2)
C1—C6—C7—N1	−174.55 (19)	N1—N2—C15—C14	175.94 (16)
C5—C6—C7—N1	4.9 (3)	C8—N2—C15—C14	0.8 (2)
N1—N2—C8—O1	3.4 (4)	C13—C14—C15—O2	1.8 (4)
C15—N2—C8—O1	177.8 (2)	C9—C14—C15—O2	−177.9 (2)
N1—N2—C8—C9	−175.91 (19)	C13—C14—C15—N2	−180.0 (2)
C15—N2—C8—C9	−1.6 (2)	C9—C14—C15—N2	0.3 (2)
O1—C8—C9—C14	−177.6 (2)		

### Hydrogen-bond geometry (Å, °)

**Table:**

Cg1 is the centroid of the C1–C6 ring.

**Table:**

*D*—H···*A*	*D*—H	H···*A*	*D*···*A*	*D*—H···*A*
C7—H7A···O1	0.93	2.18	2.857 (2)	129
C2—H2A···Cg1^i^	0.93	2.81	3.663 (2)	153

Symmetry codes: (i) −*x*+2, *y*−1/2, −*z*+3/2.
